# Evidence-based practice beliefs and implementations: a cross-sectional study among undergraduate nursing students

**DOI:** 10.1186/s12912-020-00522-x

**Published:** 2021-01-07

**Authors:** Nesrin N. Abu-Baker, Salwa AbuAlrub, Rana F. Obeidat, Kholoud Assmairan

**Affiliations:** 1 Faculty of Nursing, Community and Mental Health Nursing Department, Jordan University of Science & Technology, P.O Box 3030, Irbid, 22110 Jordan; 2 Faculty of Irbid College, Department of Applied Sciences, Al-Balqa Applied University, P.O. Box 1293, Irbid, Jordan; 3 Faculty of Nursing, Zarqa University, 247D Khawarezmi Building, Zarqa, Jordan; 4 Faculty of Nursing, Al-Albayt University, P.O Box 130040, Mafraq, 25113 Jordan

**Keywords:** Beliefs, Implementations, Evidence-based practice, Nursing students

## Abstract

**Background:**

Integrating evidence-based practice (EBP) into the daily practice of healthcare professionals has the potential to improve the practice environment as well as patient outcomes. It is essential for nurses to build their body of knowledge, standardize practice, and improve patient outcomes. This study aims to explore nursing students’ beliefs and implementations of EBP, to examine the differences in students’ beliefs and implementations by prior training of EBP, and to examine the relationship between the same.

**Methods:**

A cross-sectional survey design was used with a convenience sample of 241 nursing students from two public universities. Students were asked to answer the questions in the Evidence-Based Practice Belief and Implementation scales.

**Results:**

This study revealed that the students reported a mean total belief score of 54.32 out of 80 (*SD* = 13.63). However, they reported a much lower implementation score of 25.34 out of 72 (*SD* = 12.37). Students who received EBP training reported significantly higher total belief and implementation scores than those who did not. Finally, there was no significant relationship between belief and implementation scores (*p* > .05).

**Conclusion:**

To advance nursing science, enhance practice for future nurses, and improve patient outcomes, it is critical to teach nursing students not only the value of evidence-based knowledge, but also how to access this knowledge, appraise it, and apply it correctly as needed.

## Background

Evidence-based practice (EBP) integrates the clinical expertise, the latest and best available research evidence, as well as the patient’s unique values and circumstances [1]. This form of practice is essential for nurses as well as the nursing profession as it offers a wide variety of benefits: It helps nurses to build their own body of knowledge, minimize the gap between nursing education, research, and practice, standardize nursing practices [2], improve clinical patient outcomes, improve the quality of healthcare, and decrease healthcare costs [3]. Thus, clinical decision-making by nurses should be based on the best and most up-to-date, available research evidence [4].

Earlier studies of EBP implementation by nurses in their everyday clinical practice have shown that it is suboptimal [5–7]. Implementation of EBP is defined as its application in clinical practice [8]. Findings from previous studies indicate that nurses’ implementation of EBP can be promoted by improving their belief about EBP. Belief is the perception of the value and benefits of EBP and the perceived self-confidence in one’s knowledge and skills of EBP [8]. Nurses with a strong belief in EBP implement it more than nurses with a weak belief in the same [7, 9].

Preparing nurses for practice and ensuring that they have met a set of minimum core competencies at the point of graduation is achieved through their undergraduate education [10]. Several formal entities such as the Institute of Medicine (IOM) [4] and the Accreditation Commission for Education in Nursing (ACEN) [11] consider EBP as one of the core competencies that should be included in health care clinicians’ education. However, this does not necessarily guarantee the actual implementation of EBP in everyday clinical practice [12]. It is essential to educate undergraduate nursing students on EBP to improve their knowledge about it, to strengthen their belief regarding its benefits to patients and nurses, and to enhance their self-efficacy in implementing EBP. In order to effect this change, it is crucial to improve the education process and to focus more on the knowledge and implementation of EBP.

There is consistent evidence showing that while undergraduate nursing students hold positive beliefs about EBP and its value in patient care, they also report many challenges regarding its actual implementation in clinical practice. For instance, a mixed-methods study indicated that 118 American undergraduate nursing students found it difficult to distinguish between EBP and research. Students were able to search for evidence, but were less able to integrate evidence to plan EBP changes or disseminate best practices [13]. Additionally, a correlational study was conducted in Jordan using a sample of 612 senior nursing students. The study reported that students held positive attitudes towards research and 75% of them agreed on using nursing research in clinical practice. Students strongly believed in the usefulness of research. However, they did not believe strongly in their ability to conduct research [14]. A cross-sectional study was conducted among 188 Saudi undergraduate nursing students. Students reported positive beliefs about EBP; however, they reported a low mean score in EBP implementation (22.57 out of 72). Several significant factors have been reported as influencing EBP implementation, such as age, gender, awareness, and training on EBP [15]. A comparative survey comprised of 1383 nursing students from India, Saudi Arabia, Nigeria, and Oman. The study reported that having no authority in changing patient care policies, the slow publication of evidence, and the lack of time in the clinical area to implement the evidence were major barriers in implementing EBP according to the participating students [16].

In Jordan, evidence-based knowledge with critical thinking is one of the seven standards for the professional practice of registered nurses that were released by the Jordan Nursing Council [17]. Despite the plethora of studies on undergraduate nursing students’ beliefs about EBP and its implementation in everyday clinical practice, this topic has not been fully addressed among Jordanian undergraduate nursing students. Thus, the purpose of this study is to explore the self-reported beliefs and implementations of EBP among undergraduate nursing students in Jordan. The specific aims of this study were to (1) explore nursing students’ beliefs and implementations of EBP, (2) examine the differences in students’ beliefs and implementations by prior training of EBP, and (3) examine the relationship between nursing students’ beliefs and implementations of EBP.

## Methods

### Design and setting

A cross-sectional, correlational research survey design was used to meet the study aims. Recruitment of study participants was undertaken at two governmental universities in the northern part of Jordan. The two universities offer a four-year undergraduate nursing program aimed at graduating competent general nurses with baccalaureate degrees. The nursing research course is included as a compulsory course in the undergraduate nursing curricula in both universities.

### Population and sample

The target population of this study was the undergraduate nursing students in Jordan. The accessible population was undergraduate nursing students who are currently enrolled in the four-year BSN program in two governmental universities in the northern region of Jordan. We calculated the sample size using the G*Power software (2014). Using a conventional power estimate of 0.8, with alpha set at 0.05, and medium effect size, it was estimated that for a Pearson Correlation test, a total of 100 participants would need to be recruited to examine the relationship between the beliefs and implementations of EBP. To counteract anticipated non-response and to enhance the power of the study, 300 students were approached. The inclusion criteria of the study participants were as follows: a) senior nursing students who are in the 3^rd^or 4th-year level, b) students who are currently taking a clinical course with training in a clinical setting/hospital, c) and students who have successfully passed the nursing research course.

### Measurement

A structured questionnaire composed of two parts was used for data collection. The first part aimed to gather the demographic data of the participants: gender, age, study year level, university, and any previous EBP training received in the nursing research course. The second part contained the EBP Belief Scale and EBP Implementation scale developed by Melnyk et al. (2008) [18]. Both scales had previous satisfactory psychometric properties with a Cronbach’s alpha of more than 0.9 and good construct validity. The Evidence-Based Practice Belief Scale (EBPB) consists of 16 statements that describe the respondent’s beliefs of EBP. Students were asked to report on a five-point Likert scale their agreement or disagreement with each of the 16 statements in the scale. Response options on this scale ranged from strongly disagree (1 point) to strongly agree (5 points). All statements were positive except for two statements (statements 11 and 13), which were reversed before calculating the total and mean scores. Total scores on the EBPB ranged from 16 to 80, with a higher total score indicating a more positive belief toward EBP. In the current study, the scale showed satisfactory internal consistency reliability with a Cronbach’s Alpha of .92 for the total scale.

The Evidence-Based Practice Implementation Scale (EBPI) consists of 18 statements related to the respondent’s actual implementation of EBP in the clinical setting. Students were asked to report the frequency of the application of these statements over the past 8 weeks. The answers were ranked on a Likert scale that ranged from 0 to 4 points (0 = 0 times, 1 = 1–3 times, 2 = 4–5 times, 3 = 6–8, and 4 ≥ 8 times). The total score ranged from 0 to 72, with the higher total score indicating a more frequent utilization of EBP.

Both scales were introduced to the participating students in their original language of English because English is the official language of teaching and instruction in all schools of nursing in Jordan.

### Ethical considerations

The Institutional Review Board (IRB) at the first author’s university granted ethical approval for this study (Reference #19/122/2019). The code of ethics was addressed in the cover letter of the questionnaire. The principal investigator met the potential eligible students, provided them with an explanation about the study purpose and procedures, and gave them 5 min to read the questionnaires and to decide whether to participate in the study or not. Students who agreed to participate in the study were assured of voluntary participation and the right to withdraw from the study at any time. Questionnaires were collected anonymously without any identifying information from the participating students. The principal investigator explained to participating students that the return of completed questionnaires is an implicit consent to participate in the study. Permission to use the EBP belief scale and the EBP implementation scale for the purpose of this study was obtained from the authors of the instrument.

### Data collection procedure

After ethical approval was granted to conduct the study, data was collected during the second semester of the academic year 2018/2019 (i.e., January through June 2019). The questionnaires were distributed to the nursing students during the classroom lectures after taking permission from the lecturer. The researchers explained the purpose, the significance of the study, the inclusion criteria, and the right of the students to refuse participation in the study. Students were screened for eligibility to participate. Students who met the eligibility criteria and agreed to participate were provided with the study package that included a cover letter and the study questionnaire. Students were given 20 min to complete the questionnaire and return it to the principal investigator who was available to answer students’ questions during the data collection process.

### Data analysis

Descriptive statistics (e.g., means, standard deviations, frequencies, and percentages) were performed to describe the demographic characteristics of the participating students and the main study variables. For the belief scale, the two agreement categories (4 = agree, 5 = strongly agree) were collapsed to one category to indicate a positive belief. For the implementation scale, the three categories (2 = 4–5 times, 3 = 6–8, and 4 ≥ 8 times in the past 8 weeks) were collapsed to one category as (≥ 4 times) to indicate frequent implementation. Pearson’s correlation test was used to determine the relationship between the total scores of the EBP belief and implementation scales. A chi-square test was used to examine the difference between trained and untrained students in terms of agreement toward each EBP belief (disagreement vs. agreement) and in terms of frequency of each EBP implementation (less than 4 times vs. 4 times or more in the past 8 weeks). Finally, an independent samples *t*-test was used to examine the difference between trained and untrained students in terms of the total mean scores of EBP beliefs. The Statistical Package for Social Sciences (SPSS) software (version 22) was used for data analysis.

## Results

Among the 300 approached students, 35 students did not meet the inclusion criteria and 24 students refused to participate. Thus, a total of 241 undergraduate nursing students from both universities completed the study questionnaire for a response rate of 91%. The mean age of the participants was 22.09 years (*SD* = 1.55). The majority of the participants were females (73.4%) and in the fourth year of the undergraduate nursing program (85.1%). Further, more than half of the participants (67.6%) stated that they received EBP training before (Table 1).

**Table 1:** Distribution of the sample by demographic variables (*n* = 241)

Variables	n (%)
Gender	
Male	64 (26.6%)
Female	177 (73.4%)
Educational level	
Third-year	36 (14.9%)
Fourth-year	205 (85.1%)
University	
University A	123 (51%)
University B	118 (49%)
EBP training	
Yes	163 (67.6%)
No	78 (32.4%)
Total	241 (100%)

The total mean score of the EBP belief scale was 54.32 out of 80 (*SD* = 13.63). Overall, between 50.5 and 73.4% of students agreed or strongly agreed on the 16 statements on the EBP belief scale, which indicates positive beliefs. However, students held a more positive belief regarding the importance and the usefulness of EBP in quality patient care than in their ability to implement EBP. For example, while the majority of students believed that “EBP results in the best clinical care for patients” and that “evidence-based guidelines can improve clinical care” (73.4 and 72.2%, respectively), only about 54% of them cited that they “knew how to implement EBP sufficiently enough to make practice changes” or were “confident about their ability to implement EBP where they worked”. Students who received previous training on EBP reported more agreements (i.e., more positive beliefs) toward all items of EBP compared to those who did not receive training; however, the difference between the two groups was not always significant. For example, 60.7% of trained students believed that “they are sure that they can implement EBP” compared to 41% of untrained students χ^2^ (1, *n* = 241) = 8.26, *p* = .004. Furthermore, 58.3% of trained students were “clear about the steps of EBP” compared to 41% of untrained students χ^2^ (1, *n* = 241) = 6.30, *p* = .021 (Table 2).

**Table 2:** Responses to evidence-based practice belief scale by trained and untrained students (*n* = 241)

Your agreement to each statement:	Agree/ Strongly agree			*χ*^*2*^	*p*
	All students (*n* = 241) n (%)	Trained (*n* = 163) n (%)	Untrained (*n* = 78) n (%)		
1. “I believe that EBP results in the best clinical care for patients”.	177 (73.4%)	127 (77.9%)	50 (64.1%)	5.160	.023
2. “I am sure that evidence-based guidelines can improve clinical care”.	174 (72.2%)	121 (74.2%)	53 (67.9%)	1.038	.308
3. “I am sure that implementing EBP will improve the care that I deliver to my patients”.	167 (69.3%)	115 (70.6%)	52 (66.7%)	.374	.541
4. “I believe that critically appraising evidence is an important step in the EBP process”.	153 (63.5%)	107 (65.6%)	46 (59.0%)	1.012	0.314
5. “I believe the care that I deliver is evidence-based”.	148 (61.4%)	102 (62.6%)	46 (59.0%)	.289	.591
6. “I am sure about how to measure the outcomes of clinical care”.	152 (63.1%)	109 (66.9%)	43 (55.1%)	3.123	.077
7. “I am sure that I can implement EBP”.	131 (54.4%)	99 (60.7%)	32 (41.0%)	8.261	.004
8. “I am clear about the steps of EBP”.	127 (52.7%)	95 (58.3%)	32 (41.0%)	6.302	.021
9.” I believe that I can search for the best evidence to answer clinical questions in a time-efficient way”.	144 (59.8%)	101 (62.0%)	43 (55.1%)	1.025	.311
10. “I believe that I can overcome barriers in implementing EBP”.	124 (51.5%)	87 (53.4%)	37 (47.4%)	.745	.388
11. “I believe that EBP takes too much time”.	125 (51.9%)	80 (49.1%)	45 (57.7%)	1.567	.211
12. “I am sure that I can access the best resources in order to implement EBP”.	121 (50.5%)	83 (50.9%)	38 (48.7%)	.102	.749
13. “I believe EBP is difficult”.	142 (58.9%)	90 (55.2%)	52 (66.7%)	2.858	.091
14. “I know how to implement EBP sufficiently enough to make practice changes”.	128 (53.5%)	93 (57.1%)	35 (44.9%)	3.144	.076
15. “I am confident about my ability to implement EBP where I work”.	129 (53.5%)	96 (58.9%)	33 (42.3%)	5.836	.016
16. “I am sure that I can implement EBP in a time-efficient way”.	138 (57.3%)	99 (60.7%)	39 (50.0%)	2.485	.115

The two agreement categories (agree and strongly agree) were collapsed together to indicate positive belief

In contrast, students reported a much lower total score on the EBP implementation scale: 25.34 out of 72 (*SD* = 12.37). Less than half the students reported implementing all the listed EBPs four times or more in the last 8 weeks. For example, only about one-third of all students reported that they “used evidence to change their clinical practice”, “generated a PICO question about clinical practice”, “read and critically appraised a clinical research study”, and “accessed the database for EBP four times or more in the past eight weeks” (32.4, 33.6, 31.9, and 31.6%, respectively). The only EBP that was implemented by more than half of the students (54.8%) four times or more in the past 8 weeks was “collecting data on a patient problem”. Students who had previous training on EBP reported more frequent implementations of all listed EBPs compared to those who did not receive training; however, the difference between the two groups was not always significant. For example, 50.9% of trained students reported that they “shared an EBP guideline with a colleague” four times or more in the past 8 weeks compared to 30.8% of untrained students χ^2^ (1, *n* = 241) = 8.68, *p* = .003. Almost 50 % of the trained students “shared evidence from a research study with a patient/family member” four times or more in the past 8 weeks, compared to 28.2% of the untrained students χ^2^ (1, *n* = 241) = 9.95, *p* = .002 (Table 3).

**Table 3:** Responses to evidence-based practice implementation scale by trained and untrained students (*n* = 241)

How often each item has applied to you in the past 8 weeks:	4 times or more			*χ*^*2*^	*p*
	All students (*n* = 241) n (%)	Trained (*n* = 163) n (%)	Untrained (*n* = 78) n (%)		
1. “Collected data on a patient problem”.	132 (54.8)	95 (58.3)	37 (47.4)	2.505	.113
2. “Evaluated a care initiative by collecting patient outcome data”.	106 (44.0)	85 (52.1)	21 (26.9)	13.624	.000
3. “Used evidence to change my clinical practice”.	78 (32.4)	59 (36.2)	19 (24.4)	3377	.066
4. “Critically appraised evidence from a research study”.	85 (35.3)	64 (39.3)	21 (26.9)	3.519	.061
5. “Generated a PICO question about my clinical practice”.	81 (33.6)	62 (38.0)	19 (24.4)	4.423	.035
6. “Informally discussed evidence from a research study with a colleague”.	102 (42.3)	77 (47.2)	25 (32.1)	4.985	.026
7. “Shared evidence from a study/ies in the form of a report or presentation to *>* 2 colleagues”.	111 (46.1)	78 (47.9)	33 (42.3)	.653	.419
8. “Read and critically appraised a clinical research study”.	77 (32.0)	57 (35.0)	20 (25.6)	2.111	.146
9. “Shared an EBP guideline with a colleague”.	107 (44.4)	83 (50.9)	24 (30.8)	8.678	.003
10. “Shared evidence from a research study with a patient/family member”.	103 (42.7)	81 (49.7)	22 (28.2)	9.954	.002
11. “Shared evidence from a research study with a multidisciplinary team member”.	96 (39.8)	67 (41.1)	29 (37.2)	.339	.560
12. “Evaluated the outcomes of a practice change”.	112 (46.5)	81 (49.7)	31 (39.7)	2.099	.147
13. “Accessed the Cochrane database of systematic reviews”.	86 (35.7)	65 (39.9)	21 (26.9)	3.857	.050
14. “Accessed (A database for EBP)”	76 (31.6%)	58 (35.6)	18 (23.1)	3.821	.051
15. “Used an EBP guideline or systematic review to change clinical practice where I work”.	93 (38.6%)	68 (41.7)	25 (32.1)	2.080	.149
16. “Shared the outcome data collected with colleagues”.	101 (42.0)	76 (46.6)	25 (32.1)	4.603	.032
17. “Promoted the use of EBP to my colleagues”.	89 (36.9)	62 (38.0)	27 (34.6)	.265	.607
18. “Changed practice based on patient outcome data”.	101 (41.9)	74 (45.4)	27 (34.6)	2.520	.112

The three categories (4–5 times, 6–8 times, and 4 ≥ 8 times) were collapsed together as (≥ 4 times) to indicate frequent implementation

There was a significant difference between students’ total scores on the EBP belief scale with respect to previous training on EBP. Students who received previous training on EBP had a significantly higher mean score on the EBP belief scale compared to students who did not receive previous training on EBP (*t* (239) = 2.04, *p* = .042). In addition, there was a significant difference in the total score of EBP implementation by previous training on EBP. Students who received previous training on EBP had a significantly higher mean score on the EBP implementation scale compared to students who did not receive previous training on EBP (*t* (239) = 3.08, *p* = .002) (Table 4).

**Table 4:** Independent samples t-test between students who received EBP training and students who did not eeceive EBP training in terms of beliefs and implementations of EBP (*n* = 241)

Variable	Trained students Mean(SD)	Untrained Mean(SD)	t	df	p	Mean difference
The total score of beliefs	55.55 (13.29)	51.74 (14.06)	2.04	239	.042	3.80
The total score of implementations	27.01 (12.61)	21.86 (11.14)	3.08	239	.002	5.15

Finally, results of the Pearson correlation test revealed that there was no significant association between the total score of the EBP belief scale and the total score of the EBP implementation scale (*r* = 0.106, *p* = 0.101).

## Discussion

This study aimed to explore the self-reported beliefs regarding and implementation of EBP among undergraduate nursing students in Jordan. It is observed that Jordanian undergraduate nursing students valued EBP and its importance in delivering quality patient care as over 70% of them believed that EBP results in the best clinical care for patients and that evidence-based guidelines can improve clinical care. However, a lower percentage of students believed in their ability to implement EBP where they worked and an even lower percentage of them actually implemented EBP frequently in their everyday clinical practice. For illustration, only one-third of the students accessed a database for EBP, have read and critically appraised a clinical research study, or used evidence to change their clinical practice four times or more in the last 8 weeks. Our results are consistent with previous studies among Jordanian nursing students which also showed students had positive attitudes towards research and its usefulness to providing quality patient care but had insufficient ability to utilize research evidence in clinical practice [14]. Further, a recent study has shown that nursing students in Jordan had low knowledge about EBP regardless of their admitting university [19]. These results indicate that there could be a gap in the education process of undergraduate nursing students in Jordan about EBP. Thus, schools of nursing in Jordan have to critically review their current educational strategies on EBP and improve it to enhance students’ knowledge of EBP as well as their abilities to implement evidence in clinical practice.

The results of the current study revealed that despite the positive beliefs of the nursing students, their implementation of EBP was very low. There was no significant relationship between the total score of EBP belief and the total score of EBP implementation. Our results are consistent with those reported among Saudi as well as American nursing students who also had positive beliefs about EBP but implemented it less frequently in their everyday clinical practice [13, 15]. Moreover, in line with previous studies which showed that training on EBP was one of the significant predictors of beliefs and implementation [15], students who previously received EBP training had significantly higher total belief and implementation scores than those who did not, in this study. This finding is expected as EBP training has been shown to improve knowledge, self-efficacy in implementation, and by extension, implementation practices among nurses and nursing students [20–22]. On the other hand, in this study, we asked students whether they have received training on EBP during the nursing research course taught at their universities. More than one-third of participating students in our study cited that they had not received previous training on EBP even though all of them have successfully passed the nursing research course offered at their universities. One possible explanation for this finding could be that there is an inconsistency in the way the nursing research course is taught. It seems that EBP practice is not always included in the content taught in this course. Thus, nursing schools in Jordan have to revise their curricula to ensure that EBP is included and is taught to all students before graduation.

The results of the current study have several international implications that involve academic education and nursing curricula. There is a pressing need to enhance the education process and to focus more on the knowledge and skills of EBP. Incorporating EBP into the nursing curricula, especially the undergraduate program is critical as it is the first step to prepare the students for their professional roles as registered nurses. Sin and Bliquez (2017) stated that creative and enjoyable strategies are fundamental in order to encourage students’ commitment to and learning about EBP [23]. One of these effective strategies is teaching the EBP process by asking a clinical question, acquiring and searching for evidence, appraising then applying this evidence, and finally evaluating the effectiveness of its application in clinical practice [8]. A thematic review study demonstrated that various interactive teaching strategies and clinically integrated teaching strategies have been emphasized to enhance EBP knowledge and skills [24].

Gaining knowledge about undergraduate nursing students’ beliefs and their ability to implement EBP in a clinical setting is essential for nursing educators at the national and the international level. This knowledge might help them to evaluate and improve the current strategies utilized to educate undergraduate students about EBP. Furthermore, academic administrators and teachers should design their courses to apply EBP concepts. They should promote EBP training courses, workshops, and seminars. For example, the research course should focus more on this topic and should include clinical scenarios that involve the application of EBP. In addition, clinical courses should include assignments for the purpose of integrating EBP within their clinical cases. The scale used in this study could be implemented in clinical courses to evaluate students’ practical skills concerning EBP. Finally, nursing instructors, leaders, and practitioners should always update their EBP knowledge and skills through continuous education and workshops. Since they are the role models and instructors, they should be competent enough to teach and evaluate their students. They should also cooperate to facilitate the implementation of EBP in clinical settings to overcome any barrier.

### Study limitations and recommendations

This study sheds light on the existing gap between the belief in and the implementation of EBP among nursing students. However, convenience sampling, using two universities only, and self-report bias are all limitations of this study. In addition, the researchers did not investigate the type of EBP training that was received by the students in this study. More studies are needed in Jordan and the Middle Eastern region about EBP using larger random samples in different settings. It is also recommended to investigate the barriers that prevent nursing students from implementing EBP other than not receiving training on it. Furthermore, conducting qualitative studies might help examine and understand students’ perceptions as well as provide suggestions to bridge the gap between education and practice. Finally, future experimental studies are needed to test the effect of certain interventions on enhancing the implementation of EBP among nursing students.

## Conclusion

Evidence-based practice is essential for nursing students worldwide. However, having strong beliefs about EBP and its benefits does not necessarily mean that it is frequently implemented. On the other hand, providing training courses on EBP is an essential step in the enhancement of EBP implementation. This means that in order to advance nursing science and enhance nursing care for future nurses, it is vital to incorporate EBP within the nursing curricula. It is also critical to teach nursing students the value of evidence-based knowledge as well as how to access this knowledge, appraise it, and apply it correctly as needed. This can be achieved through rigorous cooperation between nursing administrators, clinicians, teachers, and students to enhance the implementation process.

## Data Availability

Data are available from the corresponding author upon reasonable request and with permission of Jordan University of Science and Technology.

## Abbreviations

EBP: Evidence-Based Practice
IOM: Institute of Medicine
ACEN: Accreditation Commission for Education in Nursing
EBPB: Evidence-Based Practice Belief Scale
EBPI: Evidence-Based Practice Implementation Scale
SPSS: The Statistical Package for Social Sciences
